# Production of Cloned Pigs with Targeted Attenuation of Gene Expression

**DOI:** 10.1371/journal.pone.0064613

**Published:** 2013-05-30

**Authors:** Vilceu Bordignon, Nayla El-Beirouthi, Bernardo G. Gasperin, Marcelo S. Albornoz, Mario A. Martinez-Diaz, Carine Schneider, Denyse Laurin, David Zadworny, Luis B. Agellon

**Affiliations:** 1 Department of Animal Science, McGill University, Quebec, Canada; 2 School of Dietetics and Human Nutrition, McGill University, Quebec, Canada; Leiden University Medical Center, The Netherlands

## Abstract

The objective of this study was to demonstrate that RNA interference (RNAi) and somatic cell nuclear transfer (SCNT) technologies can be used to attenuate the expression of specific genes in tissues of swine, a large animal species. Apolipoprotein E (apoE), a secreted glycoprotein known for its major role in lipid and lipoprotein metabolism and transport, was selected as the target gene for this study. Three synthetic small interfering RNAs (siRNA) targeting the porcine apoE mRNA were tested in porcine granulosa cells in primary culture and reduced apoE mRNA abundance ranging from 45–82% compared to control cells. The most effective sequence was selected for cloning into a short hairpin RNA (shRNA) expression vector under the control of RNA polymerase III (U6) promoter. Stably transfected fetal porcine fibroblast cells were generated and used to produce embryos with in vitro matured porcine oocytes, which were then transferred into the uterus of surrogate gilts. Seven live and one stillborn piglet were born from three gilts that became pregnant. Integration of the shRNA expression vector into the genome of clone piglets was confirmed by PCR and expression of the GFP transgene linked to the expression vector. Analysis showed that apoE protein levels in the liver and plasma of the clone pigs bearing the shRNA expression vector targeting the apoE mRNA was significantly reduced compared to control pigs cloned from non-transfected fibroblasts of the same cell line. These results demonstrate the feasibility of applying RNAi and SCNT technologies for introducing stable genetic modifications in somatic cells for eventual attenuation of gene expression in vivo in large animal species.

## Introduction

There is a need for large animal models to study physiopathological processes and to evaluate new therapeutics for use in humans [1]–[3]. In this regard the ideal model should closely represent human anatomy, physiology, metabolism and pathological processes. Other important considerations include the size, lifespan, availability and costs to produce and maintain. Because swine fulfill most of these conditions, there has been increasing interest to develop swine as models for biomedical research. For instance, both transgenic and non-transgenic pigs have been used as models in many fronts including surgery, nutrition, metabolism, xenotransplantation, and cell and tissue regeneration [2], [4].

Recent developments in somatic cell nuclear transfer technology has allowed for the production of clones of several large animal species [5], [6]. In particular, this technology has led to the ability to produce cloned animals from the genome of somatic cells maintained in tissue culture. The ability to introduce changes to the genome of cultured somatic cells paves a way to create specific genetic modifications that are important not only for trait improvement but also to study the pathogenesis of disease. The use of SCNT technology has already contributed to the creation of genetically engineered swine [7]–[9].

Since its discovery in studies with *C. elegans* [10], RNAi has emerged as one of the most powerful tools for studying biology [11], [12] as well as for development of new therapeutics and potential clinical applications [13]–[15]. This approach has also been used to produce transgenic mice [16], [17], rats [18], as well as livestock including swine [19]–[23].

In this study, our objective was to determine if the combination of SCNT and RNAi technologies is a viable approach to modify the expression of genes in large animal species. Here we show the attenuation of APOE gene expression in swine, *Sus scrofa*. The APOE gene was selected as the prototype gene for proof-of-concept because the protein it encodes is a secreted protein, and the resulting animal model is of potential importance in the field of atherosclerosis research.

## Results

### ApoE Knockdown in Porcine Granulosa Cells with Synthetic siRNAs

**Figure 1:**
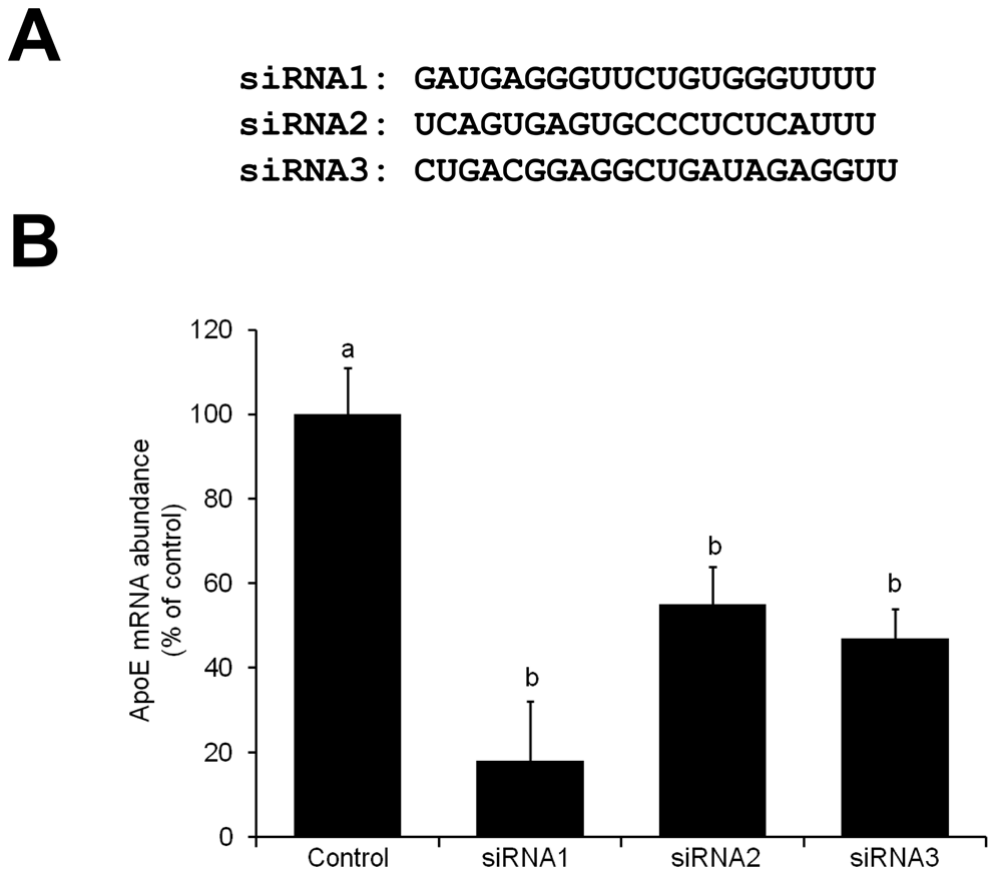
ApoE knockdown with synthetic siRNAs in cultured porcine cells. (A) Synthetic siRNAs sequences (siRNA1, siRNA2 and siRNA3) targeting the porcine apoE mRNA. (B) Effect of the siRNAs on apoE transcripts levels in cultured porcine granulosa cells. The siRNAs were introduced into the cells by lipofection. Control cells were treated with the lipofection agent alone. Cells were harvested 48 h after treatment and apoE mRNA levels were analyzed by qRT-PCR. Values were normalized to the abundance of GAPDH mRNA. The inhibitory effect of each siRNA was compared to the control group. Values are shown as percent of the control value, as the means ± SEM (n = 3 replicates). Bars that do not share a common superscript are statistically different (*P*<0.05).

In order to elect an efficient interfering RNA sequence to knockdown APOE gene expression, three siRNAs (siRNA1, siRNA2 and siRNA3; Figure 1A) targeting different regions of the porcine apoE mRNA were tested. Since fibroblasts do not express the APOE gene, granulosa cells, which are known to express the APOE gene [24], were used to validate the three siRNAs. The apoE mRNA abundance in cells treated with the three different siRNAs was different than in control cells (*P*<0.001). This indicated that the tested siRNA successfully triggered apoE transcripts cleavage in cultured porcine granulosa cells. The highest efficiency of apoE knockdown was evident with the siRNA1 sequence (82%), which was significantly superior to the siRNA3 (53%) and the siRNA2 (45%) sequences (Figure 1B).

### Stable Transfection of shRNA-expressing Vectors into Fibroblast Cells

**Figure 2:**
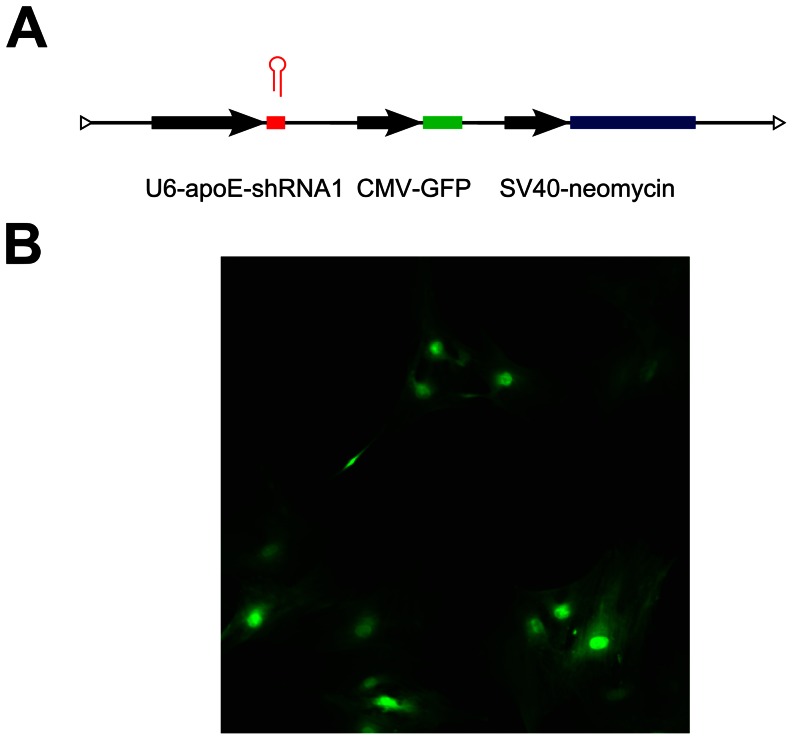
Structure and integration of the apoE-shRNA1 expression vector in transfected porcine fibroblasts. (A) The expression of the apoE-shRNA1 sequence is under the control of the U6 promoter and linked to GFP and neomycin resistance markers. (B) GFP expression in surviving cells that were selected for neomycin resistance indicating the stable integration of the apoE-shRNA1 expression vector.

Based on the above results, a shRNA-expressing vector was designed and constructed based on the siRNA1 sequence. The topology of the shRNA1 expressing vector is depicted in Figure 2A. The apoE-shRNA1 expressing vector was then used to generate stable-transfected porcine fetal fibroblast cells (Figure 2B).

### Production of Cloned Embryos Harboring the apoE-shRNA1 Expressing Vector

**Figure 3:**
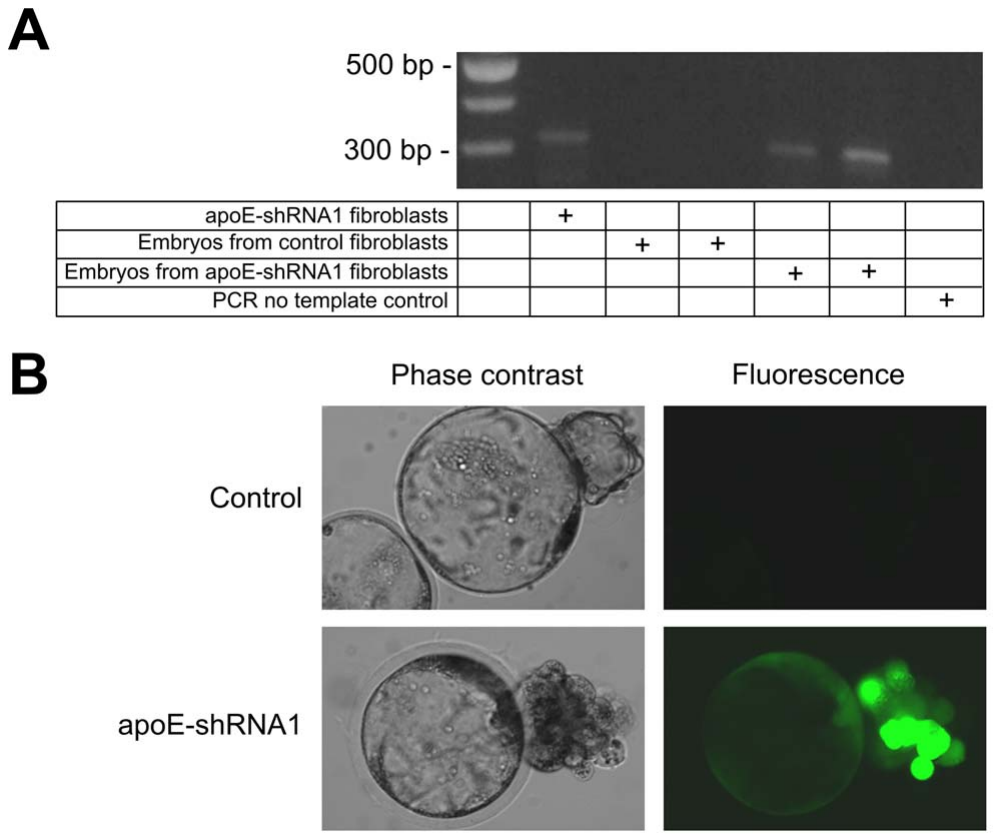
Presence of the apoE-shRNA1 and GFP expression in cloned embryos. (A) PCR detection of the apoE-shRNA1 sequence in genomic DNA purified from apoE-shRNA1 transfected fibroblasts, embryos cloned from control fibroblasts and apoE-shRNA1 fibroblasts. The DNA size marker ladder is shown in the leftmost lane of the electrophoretogram. (B) Representative images of control and apoE-shRNA1 cloned embryos at day 6 after nuclear transfer showing GFP expression viewed using phase-contrast optics (left) or the same field under fluorescence illumination (right).

In order to test whether the transfected cells would support the production of transgenic pigs with reduced expression of the APOE gene, the development in vitro of embryos produced by SCNT was first assessed. Embryo development to cleavage (72.4% and 74.6%) and blastocyst stage (34.2% and 37.2%) were similar between embryos reconstructed with non-transfected and transfected fibroblast cells from the same parental cell line, respectively (n = 1100 vs. n = 753 cases). The presence of the apoE-shRNA1 vector in the developing cloned embryos was confirmed by PCR (Figure 3A) and GFP detection by epifluorescence (Figure 3B) which was encoded by the GFP gene contained in the parental expression vector (Figure 2A).

### Production of Cloned Pigs from shRNA1 Transfected Fibroblast Cells

**Figure 4:**
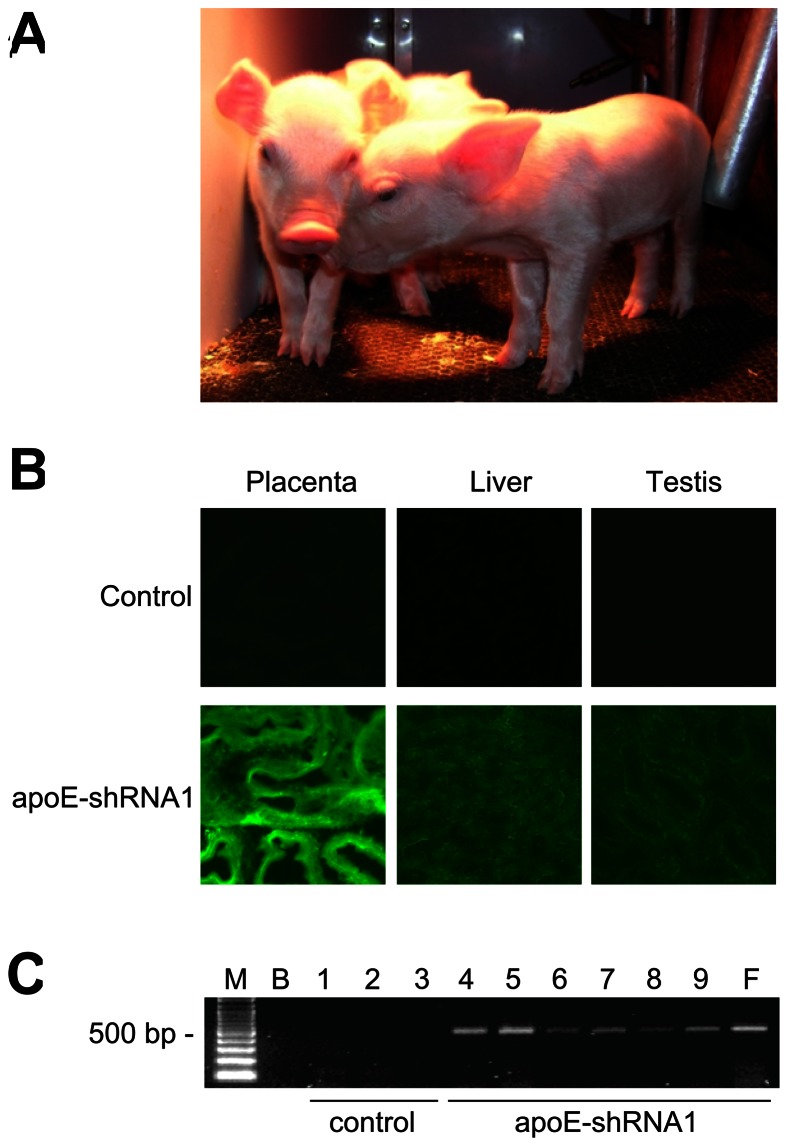
Integration and functionality of exogenous DNA in transgenic cloned pigs. (A) Cloned piglets produced from apoE-shRNA1 transfected fibroblast cells. (B) Epifluorescence images showing GFP expression in the placenta, liver and testes of pigs cloned from control cells (top row) and apoE-shRNA1 fibroblasts (bottom row). (C) PCR detection of GFP-coding sequences in liver DNA from control (lanes 1–3) and apoE-shRNA1 (lanes 4–9) cloned pigs. M, DNA size markers; B, PCR no template control; F, apoE-shRNA1 fibroblast DNA template.

The transfer of 284 cloned embryos reconstructed from transfected fibroblasts to five recipient gilts resulted in the birth of 8 cloned piglets from 3 gilts (4, 2 and 2 piglets, respectively). One of the piglets (1 of 4) from one of the recipient gilt was stillborn. The remaining 7 piglets were healthy and had normal morphology at birth and weaning (Figure 4A). One piglet from another recipient sow died of a respiratory infection after weaning at age of 4 weeks. All the other surviving clone pigs had normal growth and were healthy until they were euthanized. GFP was detected in tissue samples collected from all the apoE-shRNA1 transgenic cloned pigs but not in tissues of control cloned pigs (Figure 4B). This indicated the stable integration of the apoE-shRNA1 vector and transgene expression in the tissues of the transgenic pigs. PCR analysis of genomic DNA extracted from liver samples confirmed the presence of the apoE-shRNA1 vector in the genome of pigs cloned from apoE-shRNA1 fibroblasts cells but not in the genome of pigs cloned from non-transfected control fibroblasts (Figure 4C).

### Detection of apoE Protein in the Cloned Pigs

**Figure 5:**
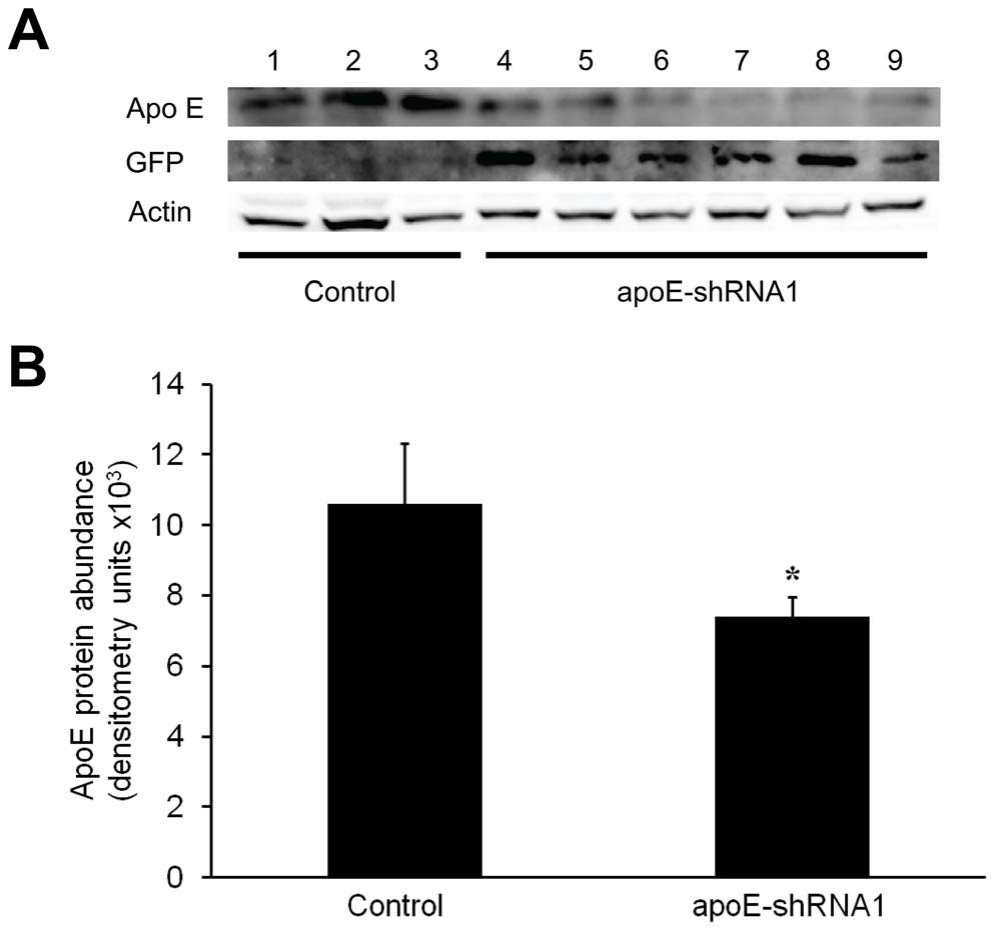
Detection of apoE and GFP proteins in control and transgenic (apoE-shRNA1) cloned pigs. (A) Immunoblots showing protein bands for apoE, GFP and β-actin in liver samples of control (lanes 1–3) and transgenic (4–9) cloned pigs. (B) Differences in apoE abundance between control and transgenic (apoE-shRNA1) liver samples was assessed by densitometric analysis. The intensity (mean ± SEM) of the apoE bands was normalized to the intensity of corresponding β-actin bands. Mean band intensity between groups was compared by ANOVA (*P = 0.05).

**Figure 6:**
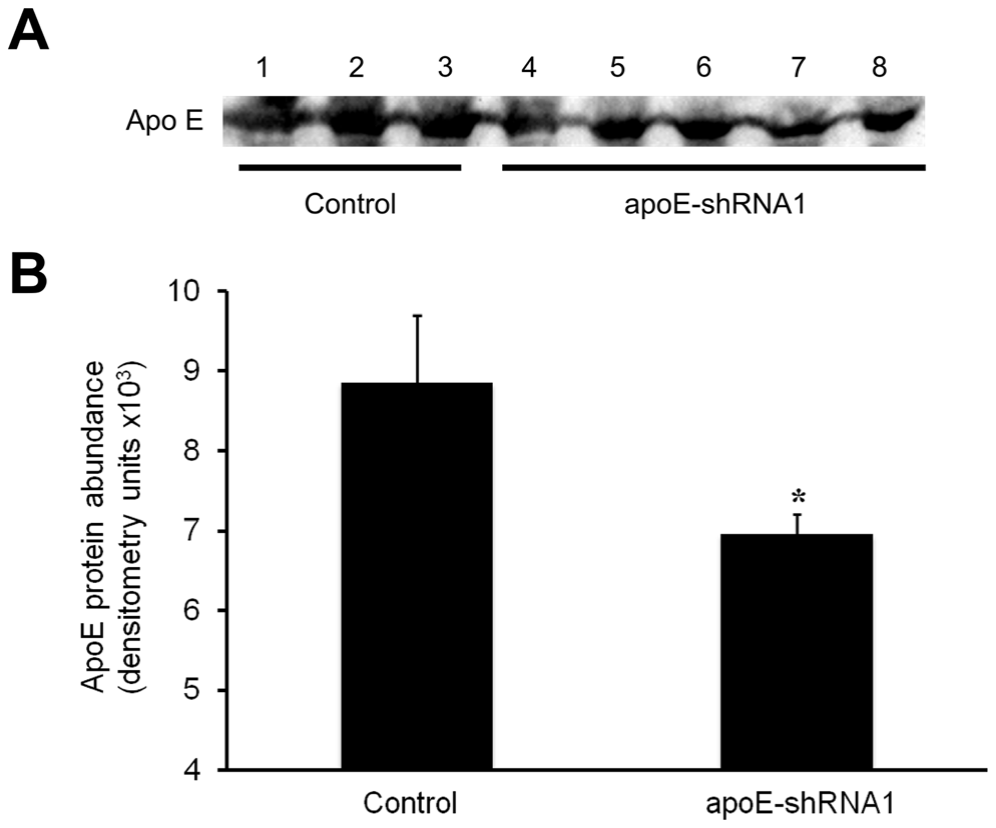
Detection of apoE in the plasma of control and transgenic (apoE-shRNA1) cloned pigs. (A) Immunoblots showing the detection of apoE protein in plasma of control (lanes 1–3) and apoE-shRNA1 transgenic (lanes 4–8) cloned pigs. (B) The intensity (mean ± SEM) of the apoE bands in equal volumes of plasma samples was assessed by densitometric analysis. Mean band intensity between groups was compared by ANOVA. Mean band intensity between groups was compared by ANOVA (*P<0.05).

In order to assess whether the presence of the apoE-shRNA1 vector affected the levels of the apoE protein, liver and plasma samples collected from the transgenic clone pigs and control clone pigs were analyzed. ApoE protein was detected in all liver and plasma samples from both control and transgenic clone pigs (Figures 5A and 6A). However, densitometric analysis of the protein bands after immunoblotting revealed lower levels of apoE in both liver (Figure 5B) and plasma (Figure 6B) of cloned transgenic pigs as compared to control pigs. Immunoblot analyses of liver samples using an anti-GFP antibody confirmed that GFP was highly expressed in transgenic pigs (Figure 5A).

## Discussion

There is great promise in the use of genetically-modified swine to improve our understanding of biology and diseases. Indeed, because swine are anatomically and physiologically similar to humans, the alteration of specific swine genes can provide ideal animal models to study the causes and potential therapeutics of genetic disorders affecting humans [2]. The swine genome is now sequenced and will facilitate the design and creation of genetically-altered swine models [25]. However, in order to enable the adoption of swine models in biomedical applications, the methods of gene manipulation as well as in the technologies used to produce gene-altered pigs require further refinements to improve efficiency, precision and simplicity. Therefore, the primary goal of this study was to determine the feasibility of using RNAi to modify gene expression in tissues and plasma of cloned pigs.

RNAi is a natural gene silencing mechanism triggered by double stranded RNA, which is highly conserved among different species [26]. The fact that stable gene silencing can be achieved by short hairpin RNAs (shRNA) expressed from DNA vectors via polymerase III promoters [27]–[29] has provided an appealing alternative to the conventional methods for gene targeting in animals [17], [18], [20], [21]. The shRNA consists of a sense and antisense small interfering RNA (siRNA) sequences linked by a non-complementary loop sequence. Upon expression, the loop is cleaved by the RNAi cell machinery to produce a functional interfering RNA molecule. Selection markers, such as GFP and antibiotic-resistance genes, can be included in the DNA vectors to enable detection and selection of cells with chromosomal integration of engineered transgenes [28], [29]. In this study, we first confirmed that small interfering RNA targeting apoE mRNA sequences decreased apoE mRNA abundance in porcine granulosa cells, which are known to express the APOE gene [24], before introducing the shRNA expressing transgene in fibroblasts, which do not express the APOE gene.

Along with SCNT, the expression of shRNAs has the potential to greatly facilitate the production of transgenic animals particularly in species, such as large domestic animals, where pluripotent stem cells are not available or still not fully characterized. To date, SCNT has been applied to clone animals of more than 20 different species. However, animal cloning efficiency from somatic cells is very low, generally less than 5% of the embryos produce by SCNT develop into live offspring [6], [30], [31]. There is overall agreement that defective epigenetic reprogramming is the main constraint affecting SCNT efficiency [30], [32]–[37]. In fact, treatments that enhance epigenetic reprogramming have been shown to improve the development of SCNT embryos [38]–[41]. Nevertheless, this technology has been successfully applied for a variety of reasons such as to create copies of elite animals with desirable phenotypic traits, to rescue deceased or endangered animals, and to produce transgenic animals. Importantly, SCNT has allowed the production of transgenic large animal species [6], [42], [43]. Indeed, the overall efficiency of transgenic livestock production by DNA pronuclear microinjection is considerably lower at <1% [44], whereas all cloned animals produced by SCNT from in vitro transfected cells have the transgene integrated into their genome [6], [9], [45]–[48]. The main advantages of SCNT over the other methods for transgenic production in large animals is that transgene insertion and expression can be ascertained in cultured cells prior to use for animal cloning. Our results confirm that exogenous DNA introduced into the genome of porcine fibroblasts is stably integrated and propagated in the embryo and tissues of the adult animal. Importantly, our ability to detect GFP fluorescence in the tissues of adult clone animals indicates that genes contained in the exogenous DNA remain functional. SCNT cloning efficiency in this study (2.82% of the transferred embryos develop to term) was similar to our previous results when SCNT embryos reconstructed with non-transfected fibroblast cells from the same parental cell line were transferred to recipient gilts [41]. This confirmed that the presence of exogenous DNA and expression of apoE-shRNA did not have a deleterious effect on post-implantation development of porcine SCNT embryo. The only stillborn piglet likely died during the passage through the birth canal because it was normal sized (1,080 g) and had no apparent lesions or anatomic abnormalities.

Analysis of liver tissue and plasma samples taken from all the surviving transgenic cloned pigs indicated reduced apoE protein abundance as compared to control clone pigs. However, there was an apparent variation in the abundance of apoE protein in the livers of different apoE-shRNA transgenic clone pigs. A number of conditions may account for variations in transgene expression in cloned animals. One possibility deals with the location or number of transgene copies integrated in the genome of donor cells since different lines of transfected cells were used to produce each pig clone. Another possibility is the efficiency of transgene expression in the tissues of cloned animals. Epigenetic changes occurring during embryo/fetal development have been observed [49], [50], and it is possible that in our study such changes affected the regulation and expression of the gene encoding the apoE-shRNA1 in the cloned pigs.

In summary, this study demonstrates that the combination of RNAi and SCNT technologies is a viable approach for producing strains of pigs, a large animal species that is of interest for biomedical research, with stable and propagatable genetic modifications.

## Materials and Methods

### Chemicals

Unless otherwise indicated, chemicals were purchased from Sigma-Aldrich (Oakville, ON, Canada).

### Animal Welfare

All animal procedures were approved by the Animal Care and Use Committee of McGill University, and were in compliance with the guidelines from the Canadian Council of Animal Care.

### Cell Culture

Porcine ovaries were obtained from a local abattoir (Olymel S.E.C./L.P.) and transferred to the laboratory in sterile 0.9% NaCl at 4°C, and were the source of granulosa cells used in this study. The content of follicles with diameter between 3–6 mm was aspirated, centrifuged at 1500 rpm for 5 min and cell pellet was washed 3 times in PBS. Cells were then resuspended and cultured in Dulbecco Modified Eagle Medium (DMEM) supplemented with 10% fetal bovine serum (FBS), 100 U/ml penicillin and 100 µg/ml streptomycin (Life Technologies Inc., Burlington, ON) at 38.5°C in a humidified atmosphere of 5% CO_2_ and 95% air. The culture medium was replaced every 48 h. Once reaching confluence, cells were treated with 0.125% trypsin/EDTA (Life Technologies Inc.) for 1 min at 37°C, and then subcultured in 6-well plates (Nunclon, Denmark). Only first passage cells were used for RNAi experiments.

### siRNA Testing in Granulosa Cells

Three synthetic siRNAs (siRNA1, siRNA2 and siRNA3; Figure 1A) targeting the porcine apoE mRNA (GI:311232) were designed using the siRNA Target Finder software from Ambion (Life Technologies Inc.). The sequences were confirmed for specificity using a BLAST search (www.ncbi.nlm.nih.gov/BLAST). The siRNA testing was carried out using the siPort NeoFX transfection agent according to the manufacturer’s instructions (Life Technologies Inc.). Each siRNA (10 nM) was tested in 2 wells (each containing 3×10^5^ cells) and the experiment was repeated 3 times. ApoE mRNA levels were assessed by qRT-PCR at 48 h after siRNA transfection. Cells in the control wells were incubated with the siPort NeoFX without siRNA to monitor cytotoxicity and cell death. The final concentration for cell transfection was adjusted to 10 nM/well by preparing a 2 µM stock solution of each siRNA in double distilled water followed by dilution in DMEM (10 µl of the stock in 90 µl DMEM). The siPORT NeoFX (5 µl) was diluted in DMEM (95 µl). The diluted siPORT NeoFX and the siRNAs were combined and maintained for 10 min at room temperature. A total of 200 µl was dispensed to each well of a 6-well plate and then 2 ml of DMEM containing 3×10^5^ cells was layered on top. The transfection medium was replaced with culture medium 24 h after cell transfection. The abundance of apoE mRNA was assessed by qRT-PCR 48 h post-transfection.

### RNA Extraction and PCR

Total RNA was extracted using the RNA Mini kit (QIAGEN Inc., Toronto, ON) and concentration determined by absorbance at 260 nm using a NanoDrop spectrophotometer (Thermo Fischer Scientific Inc., Wilmington, DE). RNA extracted from 2 duplicate wells of each treatment were combined and normalized to 500 ng/µl. Genomic DNA was digested using 1 U/µl of DNase I amplification grade (Life Technologies Inc.). The resulting preparation was then reverse-transcribed using 100 ng random hexamers (Amersham Biosciences Corp, Piscataway, NJ) and 200 units of SuperScript II reverse transcriptase (Life Technologies Inc.) and cDNA was stored at −20°C. All DNA primers (Table 1) were ordered from Life Technologies Inc and used following the PCR profile: 94°C, 2 min; 40×(94°C; 30 s; 60°C, 25 s; 72°C, 30 s); 72°C; 10 min; 4°C. PCR products were separated on 1.5% agarose gels and visualized by ethidium bromide staining. All qRT-PCR assays were conducted in a 96 well PCR plate using the ABI 7500 thermal cycler (Applied Biosystems, Foster City, CA) and the SYBR® GreenER™ Two-Step qRT-PCR Universal Kit (Life Technologies Inc.) in 10 µl total volume. All primers were designed spanning 2 exons (Table 1) and melting-curve analyses were done to verify product identity. Triplicate samples of each template were analyzed for apoE mRNA quantitation while duplicates were used for glyceraldehyde 3-phosphate dehydrogenase mRNA which was used as the internal reference.

**Table 1:** Sequence of oligonucleotide primers.

Primer name	Primer sequence (5′→3′)	Annealing temperature	Amplicon size (bp)
apoE.F1 apoE.R1	GGCCGCTTCTGGGATTAC CCTTCACCTCCTTCATGCTC	60°C	133
cyclo.F1 cyclo.R1	ACCGTCTTCTTCGACATCGC CTTGCTGGTCTTGCCATTCC	62°C	550
gapdh.F2 gapdh.R2	CAGCAATGCCTCCTGTACCA GATGCCGAAGTTGTCATGGA	60°C	92
pRNA.F pRNA.R	TACGATACAAGGCTGTTAGAGAG TAGAAGGCACAGTCGAGG	60°C	329
GFP.F GFP.R	TCTGCACCACCGGCAAGCTG TTGGACAGGGCGCTCTGGGT	60°C	487

### Construction of the apoE-shRNA1 Expression Vector

Two complementary DNA oligonucleotides (positive and negative strands) corresponding to the siRNA1 sequence (Figure 1) were synthesized (Life Technologies Inc.). The positive strand contains 19 nucleotides corresponding to the antisense strand of the siRNA1, a loop of 9 non-complementary nucleotides, followed by the sense strand of the siRNA1. A flanking sequence corresponding to the BamHI restriction site was appended at the 5′ end and a HindIII restriction site was also appended to the 5′end of the complementary strand. The single stranded DNA oligonucleotides (100 µM of each) were annealed in a solution containing 3 M NaCl, 0.3 M sodium citrate (pH 7). The annealing process consisted of a denaturation step at 95°C for 5 min followed by 80°C for 10 min and then by the gradual decrease of the temperature (1°C every 90 s) until reaching room temperature. The annealed DNA oligonucleotides were treated with BamHI and HindIII (New England Biolabs), and then ligated into the BamHI and HindIII treated pRNAT.U6.Neo plasmid (Genscript Corp, Piscataway, NJ) using T4 DNA ligase (New England Biolabs) at 16°C overnight. The shRNA-expressing plasmid was cultivated in *E. coli* DH5α as previously described [51]. Single colonies were first checked for presence of the shRNA1 sequence by PCR. The apoE-shRNA1 expression plasmid was purified using a QIA spin Miniprep kit (QIAGEN Inc.) and/or Pure link Hipure Plasmid Maxiprep (Life Technologies Inc.) and sequenced at the McGill University and Genome Quebec Innovation Center (http://www.gqinnovationcenter.com/index.aspx?l=e).

### Transfection of Fetal Fibroblasts Cells

A fetal fibroblast cell line established from a male porcine fetus, which was previously tested and successfully used to produce cloned pigs by SCNT in our laboratory [41], was used for cell transfection. First passage cells were transfected with the shRNA1using Lipofectamine 2000 (Life Technologies Inc.). The apoE-shRNA1 plasmid (30 µg) was incubated with 30 µl of Lipofectamine in DMEM for 20 min to allow the formation of transfection complexes, and then 20 µl were added to each 75 cm^2^ cell culture flask when cells were approximately 80–90% confluent. The DMEM free of antibiotics and serum was replaced with regular culture medium 18 h after transfection. Stably transfected cells were selected for resistance to G418 (Geneticin; Life Technologies Inc.) starting 48 h after transfection. The G418 concentration and time of treatment for cell selection was as follows: 300 µg/ml for the first 5 days, 200 µg/ml for the next 2 days, 300 µg/ml for another 5 days, and then 200 µg/ml for additional 22 days. Cells were stored frozen in DMEM supplemented with 10% DMSO and 10% FBS under liquid N_2_.

### Production of Embryos by SCNT

Porcine ovaries were collected from a local abattoir and cumulus-oocyte complexes were selected and matured in vitro for 44–46 h under standard conditions [41]. Matured oocytes with a polar body were selected and cultured in TCM199 (Invitrogen, Life Technologies Inc.) supplemented with 0.4 µg/ml demecolcine and 0.05 M sucrose for 40–60 min. Oocytes were then transferred to Tyrode’s Lactate-Pyruvate- HEPES medium supplemented with 7.5 µg/ml cytochalasin B for 5–10 min and were then enucleated by removing the oocyte chromatin together with the first polar body. A transfected fibroblast cell was transferred into the perivitelline space of each enucleated oocyte and electrically fused using a single DC pulse of 1.6 kV/cm for 70 µsec. Electrofusion was performed in a 0.28 M D-mannitol solution supplemented with 50 µM CaCl_2_, 100 µM MgSO_4_, and 0.1% polyvinyl alcohol [41]. Reconstructed oocytes were cultured in porcine zygote medium (PZM-3) supplemented with 3 mg/ml bovine serum albumin for 1 h and then activated using ionomycin (15 µM/5 min) followed by exposure to strontium chloride (10 mM/4 h) in PZM-3 without calcium [52]. After activation, embryos were cultured in PZM-3 in a humidified atmosphere of 5% CO_2_ and 95% air at 38.5°C for 5–6 days.

### Embryo Transfer and Production of Cloned Pigs

Embryos that developed to morula and blastocyst stages after 5–6 days in culture were briefly examined under a fluorescent microscope to confirm GFP expression and were then transferred into the uterus of estrus synchronized recipient gilts. Control cloned pigs were produced from embryos reconstructed using non-transfected fibroblasts cells of the same parental cell line. Gilts with body weights between 105–115 kg were used as recipients for embryo transfer. The recipient gilts (n = 5) were prepared by daily oral administration of the active synthetic progestin, altrenogest (20 mg/day; Regu-Mate®, Intervet Canada Corp., Kirkland, QC) for 12 or 13 days, followed by 1000 IU eCG (Folligon®, Intervet Canada) injected in the last day of altrenogest treatment and 500 IU hCG (Chorulon®, Intervet Canada) 72 h later. Embryos were transferred 6 days after hCG injection. Pregnancy diagnosis was performed by ultrasonography between days 20 and 25 after embryo transfer and the pregnant females were monitored monthly with ultrasound until parturition. Parturition was induced by injecting PGF_2α_ (10 mg dinoprost tromethamine; Lutalyse®, Pfizer Canada Inc., Kirkland QC, Canada) at day 115 of pregnancy.

### Detection of Vector Integration in Cloned Embryos and Tissues of Cloned Piglets

Single embryos were digested with 10 µg proteinase K (QIAGEN Inc.) in 10 µl of double distilled dH_2_O with 1×PCR buffer at 56°C overnight. Genomic DNA was subjected to conventional PCR using the vector primers pRNA.F and pRNA.R (Table 1). The PCR product, a 329 bp amplicon, was detected by gel electrophoresis to confirm the presence of the apoE-shRNA1 expressing vector in the genome of the developing cloned embryos. Genomic DNA was extracted from tissues of cloned pigs using the Maxwell 16 System (Promega, Madison, WI) and PCR amplification was performed with primers pRNA.F and pRNA.R or GFP-F and GFP-R (Table 1). For verification of GFP expression, tissues were frozen and stored in liquid nitrogen, and then 10 µm cryocuts prepared in a Shandon Cryotome E (Thermo Fischer Scientific Inc.) were mounted on glass slides and evaluated using an epifluorescence microscope.

### Immunodetection of apoE and GFP in the Cloned Pigs

Liver and blood samples were collected from the transgenic and control animals. Three cloned pigs produced from non-transfected fibroblasts of the same cell line that were raised in similar conditions were used as controls for tissue and blood analyses. Proteins were extracted from liver samples (∼5 mg) using total extraction buffer and concentration was determined in a NanoDrop spectrophotometer. After heating the samples at 95°C for 5 min, proteins (30 µg) were subjected to 12% SDS gel and then electrotransferred onto nitrocellulose membranes. After blocking for 2 h with 5% skim milk in PBS containing 0.1% Tween-20 (PBS-T), blots were incubated overnight at 4°C with 1∶1000 diluted goat anti-human apoE (sc-31821; Santa Cruz Biotechnology Inc., Santa Cruz, CA) or 1∶5000 diluted rabbit anti-human β actin (ab8227; Abcam, Cambridge, MA) with agitation, followed by three washes (10 min each) with PBS-T. The blots were then incubated with 1∶5000 diluted donkey anti-goat IgG-HRP (sc-2020; Santa Cruz Biotechnology Inc.) or 1∶5000 diluted goat anti-rabbit IgG-HRP (ab6721; Abcam) for 2 h with agitation, followed by three washes (10 min each) with PBS-T. To detect apoE levels in the plasma of control and transgenic clone pigs, samples (8 µl; ∼500 µg of total plasma protein) were subjected to 12% SDS gel and electrotransferred onto nitrocellulose membranes. After blocking, the blot was incubated overnight with 1∶1000 diluted goat anti-human apoE (sc-31821; Santa Cruz Biotechnology Inc.). The blot was then incubated with 1∶5000 diluted donkey anti-goat IgG-HRP (sc-2020; Santa Cruz Biotechnology Inc.). All the blots were incubated in SuperSignal West Femto Maximum Sensitivity Substrate (Thermo Fischer Scientific Inc.) for 3 min and visualized using the ChemiDoc system (Bio-Rad, Mississauga, ON). To compare apoE levels between clone and transgenic clone pigs, the band volume for each sample was assessed using the Image Lab software (Bio-Rad). For liver samples, the values for apoE band volumes were corrected to the band volume of β-actin. In plasma samples, the same amount of protein was loaded as assessed by bicinchoninic acid assay. To confirm the presence of GFP in the cloned pigs, samples of liver protein (40 µg) from each animal were boiled for 5 min and subjected to 12% SDS gel and eletrotransferred onto a nitrocellulose membrane. The membrane was blocked and then incubated overnight at 4°C with 1∶2500 rabbit anti-*Aequorea victoria* GFP (GTX20290; GeneTex Inc., Irvine, CA) diluted in PBS containing 3% bovine serum albumin. After washing, the membrane was incubated with 1∶5000 goat anti-rabbit IgG-HRP (ab6721; Abcam). Visualization of the immune complexes was conducted as described above.

### Statistical Analysis

Data were analyzed using the JMP software (SAS Institute Inc., Cary, NC). Gene silencing efficiency after siRNA treatments was analyzed by one-way ANOVA followed by Tukey–Kramer HSD. The intensity of the protein bands after immunoblotting was compared by ANOVA. Differences were considered to be statistically significant at the 95% confidence level (*P≤*0.05).
